# Mitochondrial DNA Polymerase POLG1 Disease Mutations and Germline Variants Promote Tumorigenic Properties

**DOI:** 10.1371/journal.pone.0139846

**Published:** 2015-10-15

**Authors:** Bhupendra Singh, Kjerstin M. Owens, Prachi Bajpai, Mohamed Mokhtar Desouki, Vinodh Srinivasasainagendra, Hemant K. Tiwari, Keshav K. Singh

**Affiliations:** 1 Department of Genetics, University of Alabama at Birmingham, Birmingham, AL, United States of America; 2 Department of Cancer Genetics, Roswell Park Cancer Institute, Buffalo, NY, United States of America; 3 Department of Pathology, Microbiology and Immunology, Vanderbilt University, Nashville, TN, United States of America; 4 Section on Statistical Genetics, Department of Biostatistics, University of Alabama at Birmingham, Birmingham, AL, United States of America; 5 Department of Pathology, University of Alabama at Birmingham, Birmingham, Alabama, United States of America; 6 Department of Environmental Health, University of Alabama at Birmingham, Birmingham, Alabama, United States of America; 7 Center for Free Radical Biology, University of Alabama at Birmingham, Birmingham, Alabama, United States of America; 8 Center for Aging, University of Alabama at Birmingham, Birmingham, Alabama, United States of America; 9 UAB Comprehensive Cancer Center, University of Alabama at Birmingham, Birmingham, Alabama, United States of America; 10 Birmingham Veterans Affairs Medical Center, Birmingham, AL, 35294, United States of America; University of Texas Health Science Center at San Antonio, UNITED STATES

## Abstract

Germline mutations in mitochondrial DNA polymerase gamma (POLG1) induce mitochondrial DNA (mtDNA) mutations, depletion, and decrease oxidative phosphorylation. Earlier, we identified somatic mutations in POLG1 and the contribution of these mutations in human cancer. However, a role for germline variations in POLG1 in human cancers is unknown. In this study, we examined a role for disease associated germline variants of POLG1, POLG1 gene expression, copy number variation and regulation in human cancers. We analyzed the mutations, expression and copy number variation in *POLG1* in several cancer databases and validated the analyses in primary breast tumors and breast cancer cell lines. We discovered 5-aza-2'-deoxycytidine led epigenetic regulation of *POLG1*, mtDNA-encoded genes and increased mitochondrial respiration. We conducted comprehensive race based bioinformatics analyses of *POLG1* gene in more than 33,000 European-Americans and 5,000 African-Americans. We identified a mitochondrial disease causing missense variation in polymerase domain of POLG1 protein at amino acid 1143 (E1143G) to be 25 times more prevalent in European-Americans (allele frequency 0.03777) when compared to African-American (allele frequency 0.00151) population. We identified T251I and P587L missense variations in exonuclease and linker region of POLG1 also to be more prevalent in European-Americans. Expression of these variants increased glucose consumption, decreased ATP production and increased matrigel invasion. Interestingly, conditional expression of these variants revealed that matrigel invasion properties conferred by these germline variants were reversible suggesting a role of epigenetic regulators. Indeed, we identified a set of miRNA whose expression was reversible after variant expression was turned off. Together, our studies demonstrate altered genetic and epigenetic regulation of POLG1 in human cancers and suggest a role for POLG1 germline variants in promoting tumorigenic properties.

## Introduction

The human mitochondrial genome is 16.6 kb in size and encodes for 13 polypeptides of the oxidative phosphorylation (OXPHOS) system which generates most of the cellular adenosine triphosphate (ATP) in a cell. The remaining more than 85 polypeptides required for the assembly of OXPHOS system are encoded by nuclear genome. Decreased mitochondrial OXPHOS is one of the most common phenotypes of cancer cells [1–4]. Mitochondrial DNA depletion impairs OXPHOS and adaptive metabolic responses [5]. Several decades ago, Otto Warburg described altered OXPHOS in the mitochondria of tumor cells and hypothesized that decreased OXPHOS in cancer cells led to an increased glycolytic energy production, a phenomenon known as the Warburg effect [4].

Polymerase gamma is the only DNA polymerase known to function in human mitochondria. It contains a large catalytic subunit, POLG1 (140 kDa), and two identical accessory subunits encoded by POLG2 (55 kDa) [6]. POLG1 consists of an exonuclease domain, a polymerase domain along with an intervening linker region [7]. Polymerase gamma contains DNA polymerase, 3'–5' exonuclease and 5'-deoxyribose phosphate lyase activities and is involved in replication and repair of mtDNA [8].

Mitochondrial DNA depletion syndrome (MDS) results from mutation(s) in nuclear-encoded genes that participate in mtDNA replication, mitochondrial nucleotide metabolism and in the nucleotide salvage pathway [1,9–14]. Nuclear-encoded *POLG1* is the most frequent target of gene mutation and is involved in a variety of mitochondrial diseases. Germline mutations in the *POLG1* are linked with a wide variety of mitochondrial diseases, ranging from Alpers' syndrome to male infertility, progressive external opthalmoplegia (PEO), Leigh's syndrome, Parkinsonism and other mitochondrial diseases [8,15–18]. Most disease phenotypes associated with germline mutations in *POLG1* are due to mutations and/or depletions in mtDNA.

To date, more than 250 germline and somatic mutations in *POLG1* have been identified [19–22]. Role of *POLG1* somatic mutations in human cancers have been shown by us and others [21,22]. Genetic diversity in ethnic populations arises from differences in individual bases known as variants or single nucleotide polymorphisms (SNPs). These variants may affect biological functions and potentially serve as important signature marks in association studies of individuals in a population study. Allelic frequency of SNPs varies from one population to another contributing to vast fraction of genetic diversity in populations. These SNPs would help explain the differences in genetic predisposition and susceptibility to diseases from one population to another. Although, germline polymorphic mutations/SNPs in *POLG1* have been shown to result in decreased mtDNA content, decreased OXPHOS and several human mitochondrial diseases, their role in the pathogenesis of cancer is still unclear [7,8,15–18,23,24].

In our previous study, we identified somatic mutations in *POLG1* gene in breast cancer and described their role in tumorigenesis [22]. In this study, we examined copy number variation, expression and regulation of *POLG1* gene in human cancers. We also studied *POLG1* disease associated germline variations in American population and their association with cancer. We provide evidence that altered *POLG1* expression as well as germline variations in *POLG1* gene contribute to tumorigenesis.

## Materials and Methods

### Tumor Samples

All experiments were approved by the Roswell Park Cancer Institute Institutional Review Board, permit number I1085M. Normal and matched breast tumor RNA samples were obtained from the biorepository resource facility of the Roswell Park Cancer Institute. Anonymous tissue microarray (TMA) slide from the Cooperative Human Tissue Network (CHTN) and Breast Cancer Program 1 of the National Cancer Institute, National Institutes of Health, Bethesda, MD was used for immunohistochemistry. Consent from the patients was not needed, as anonymous samples were used in the study.

### In Silico Analyses

The expression of POLG1 was analyzed between the normal and corresponding tumors among multiple cancers, such as breast, brain, bladder, cervix, colorectal, esophagus, head & neck, kidney, lung, pancreas, skin, myeloma, leukemia, and prostate cancers using Oncomine database [25]http://journals.plos.org/plosone/article?id=10.1371/journal.pone.0024792-pone.0024792-Rhodes1. Oncomine is a cancer microarray database and web-based data-mining platform that provide human cancer data from genome-wide expression analyses. *POLG1* tumor mutations and copy number variations were analyzed in several human cancers using Cosmic [20] and cBioPortal [26] databases. Cosmic database is a curated web-based database that contains somatic mutation information and related details from human cancers from peer reviewed published articles. The cBioPortal for Cancer Genomics database contains human cancer mutation, DNA copy number alterations, mRNA and microRNA expression data and is developed and maintained by Memorial Sloan-Kettering Cancer Center. The cBioPortal contains data sets from published cancer studies and more than 20 studies that are currently in The Cancer Genome Atlas (TCGA) pipeline [26]. *POLG1* germline variations were analyzed in American ethnic population using ExAC and 1000 genome databases [27].

### Cell Culture

MCF-12A, MCF-7 and MDA-MB-231 cell lines were purchased from ATCC (Manassas, VA). MCF-12A cells were grown in DMEM/F12 50/50 media (Cellgro, Herndon, VA) supplemented with 10% fetal bovine serum (Atlanta Biologicals, Lawrenceville, GA), 0.1 μg/ml cholera toxin (Sigma, St. Louis, MO), 20.0 ng/ml epidermal growth factor (PeproTech, Rocky Hill, NJ), 0.1% penicillin/streptomycin (Cellgro), 0.5 μg/ml hydrocortisone (Sigma) and 10.0 μg/ml insulin (Sigma). All other cell lines were maintained in DMEM (Cellgro) with 10% fetal bovine serum (Atlanta Biologicals) and 0.1% penicillin/streptomycin. Rho^0^ cells were grown in 50 μg/ml uridine (Sigma) supplemented media. All cells were maintained in a 37°C, 95% humidity, and 5% carbon dioxide environment. For epigenetic studies, MDA-MB-231 cells were treated with 1–10μM of 5-aza-2'-deoxycytidine (5-aza) (Sigma) for 96h. In parallel, MDA-MB-231 cells were also treated with vehicle (DMSO) only as control. For growth rate analyses of vehicle or 5-aza treated MDA-MB-231 cells after 96h of treatment, 1x10^5^ cells were seeded in 6-well dishes and the total cell numbers were counted with a hemocytometer at after every 24h up to 6 days.

### Plasmid Construction and Site-Directed Mutagenesis

Full-length *POLG1* cDNA was subcloned into the inducible mammalian expression vector, pTRE-Tight-BI-AcGFP1 (Clontech, Palo Alto, CA). E1143G (polymerase domain), T251I (linker region), P587L (exonuclease domain) and T251I and P587L (double mutant) site-directed mutants were created as described earlier [22]. Mutations were confirmed by sequencing the complete open reading frame of each mutant clone.

### Yeast Petite Formation Assay

The frequency of petite formation in the BY4741 background was determined as previously described [28]. Briefly, wild type yeast strain BY4741 was transformed with empty p426ADH vector, wild-type human POLG1 or human POLG1 mutants (E1143G, T251I, P587L and T251I/P587L double mutant) containing vector. For each set of experiments five to seven independent cultures of each transformed strain were plated onto yeast extract-peptone-dextrose (YPD) medium and grown for 3 days at 30°C to allow unequivocal identification of petite and grande colonies. Culture petite frequencies in case of wild-type human POLG1 or human POLG1 mutants were expressed as the number of petite colonies divided by the total number of colonies multiplied by 100%.

### Matrigel Invasion Assay

MCF-7 Tet-on cells were transfected with wild type or mutant POLG1 plasmid and were sorted for GFP using a FACSCalibur (Becton Dickinson Biosciences). Five days after doxycycline (dox) treatment (1000 ng/ml) or 5 days with dox treatment and then 7 days without dox, the cells were analyzed for *in vitro* Matrigel invasion. The cells were plated in serum-free media in an upper Boyden chamber with a Matrigel membrane. Complete media containing 10% fetal bovine serum were added to the bottom well as a chemoattractant. The cells in the chamber were incubated for 24h and the membrane was fixed and stained with the Diff-Quick Stain Set (Dade Behring, Newark, DE, USA). The invading cells were counted in 6 views per membrane under a microscope at 20X magnification. Cell counts were averaged and statistically analyzed.

### Mitochondrial Functional Analyses

MCF-7 Tet-On cells were transiently transfected with the pTRE-Tight-BI-AcGFP1 vector containing either POLG1 E1143G or POLG1 D1135A using the Fugene HD transfection reagent (Promega, Madison, WI) protocol. Media containing 1000 ng/ml dox were added 4h after transfection. Expression of POLG1 E1143G or POLG1 D1135A was induced by 1000 ng/ml dox for 10 days and glucose consumption and ATP levels were measured. Glucose in culture media at day 0 and at day 10 of dox treatment was measured with a OneTouch Ultra LifeScan Gluocometer (Milpitas, CA). Total cell numbers were counted with a hemocytometer at each time point. Glucose consumption was calculated as pmol of glucose/cells per well. Similarly, ATP levels in cells at day 0 and at day 10 of dox treatment was measured using CellTiter-Glo reagent (Promega) according to manufacturer's protocol. The luminescence in each well was measured using Luminoskan Ascent plate reader (Thermo Electro Corp., Waltham, MA). Total cell numbers were counted in each well with a hemocytometer at each time point and ATP level/cell was calculated. Mitochondrial respiratory activity in 5-aza treated MDA-MB-231 cells was measured by the rate of resazurin reduction as previously described [29].

### Mitochondrial DNA Content Analysis

The mtDNA content was analyzed by real-time PCR by absolute quantification with the following primers: mMitoF: 5'-CTAGAAACCCCGAAACCAAA-3', mMitoR: 5'-CCAGCTATCACCAAGCTC GT-3', mB2MF: 5'-ATGGGAAGCCGAACATACTG-3', and mB2MR: 5'-CAGTCTCAGTGGGGGTG AAT -3'. Beta-2-Microglobulin (B2M) was used as an internal control.

### Gene Expression Analyses

RNA from breast tumors and normal breast tissues was reverse transcribed using Superscript III First Strand kit (Invitrogen, Carlsbad, CA). The RNA was isolated from cell lines by Trizol extraction method according to the manufacturer's protocol (Invitrogen) and reverse transcribed using the Superscript III First Strand kit (Invitrogen). RT PCR was performed to measure the expression levels of *Polg1*, *ND2*, *ND4*, *ND5*, *Cyt b* and *COXI* genes using GoTaq Green 2X Master Mix (Promega) and gene specific primers. β-actin was used as an internal control.

### miRNA Expression Array

Total RNA samples were extracted from POLG1 D1135A expressing cells after 5 days of dox induction and from the cells grown in the absence of dox for 7 more days after 5 days of dox induction by Trizol extraction method (Invitrogen). Illumina human miRNA expression array was performed with these total RNA samples as described earlier [30].

### Spectral Karyotyping (SKY) Analysis

After mitotic arrest for 2 h with Colcemid, MDA-MB-231 and BT-549 breast cancer cells were harvested and treated with hypotonic solution according to the standard protocol as described earlier [31]. Chromosome number and chromosomal rearrangements or alterations including translocation, deletion and duplication were analyzed in these cells.

### Immunohistochemistry (IHC)

Tissue microarray (TMA) slide purchased from the Cooperative Human Tissue Network (CHTN) of the National Cancer Institute, National Institutes of Health, Bethesda, MD containing 53 (13 benign and 40 malignant) interpretable breast tissue cores in duplicate were used in the present study. The TMA contains 8 and 5 tissue cores from non-neoplastic breast tissue from patients with and without breast cancer, respectively. The TMA also contain 6, 7, 7, 6, 7 and 7 tissue cores from low grade ductal carcinoma in situ (DCIS), high grade DCIS, grade 1–2 invasive duct carcinoma (IDC), grade 3 IDC, invasive lobular carcinoma (ILC) and regional lymph nodes harboring metastatic breast carcinomas (LN met), respectively.

Sections from formalin-fixed, paraffin-embedded benign liver tissue slide were used as positive control. The immunohistochemistry protocol as described earlier was applied with modifications [32]. Briefly, the slides were de-paraffinized by incubation in xylene and ascending grades of alcohol. Antigen retrieval was done by heating in citrate-based, antigen unmasking solution (Vector Laboratories, Burlingame, CA) for 30 minutes at 98°C, incubated in 3% hydrogen peroxide (H2O2) for 10 minutes, blocked with blocking peptide for 30 minutes, incubated with 1:50 anti-POLG1 (a gift from Dr. William C. Copeland, NIEHS) antibody for one hour at room temperature, followed by incubation with biotinylated secondary antibody for 30 min and another 30 min with Vectastatin ABC kit (Vector Laboratories). Color was developed by incubating slides with peroxidase substrate solution followed by counterstaining with hematoxylin. Section from benign liver was also incubated with secondary antibody only to check nonspecific bindings. All sections were examined with Olympus BX50 microscope. The pictures were taken with Olympus DP 70 camera connected to DP Controller software (Olympus, Center Valley, PA).

Scoring of cytoplasmic immunoreactivity for POLG1 was considered to be negative or positive, with the same parameters we described earlier (score + < 10% positive, score ++ 10–50% positive and score +++ > 50% positive) [32]. The grades and IHC scores were considered nominal to find out correlation coefficient.

## Results

### POLG1 Expression Is Altered in Primary Tumors

**Fig 1:**
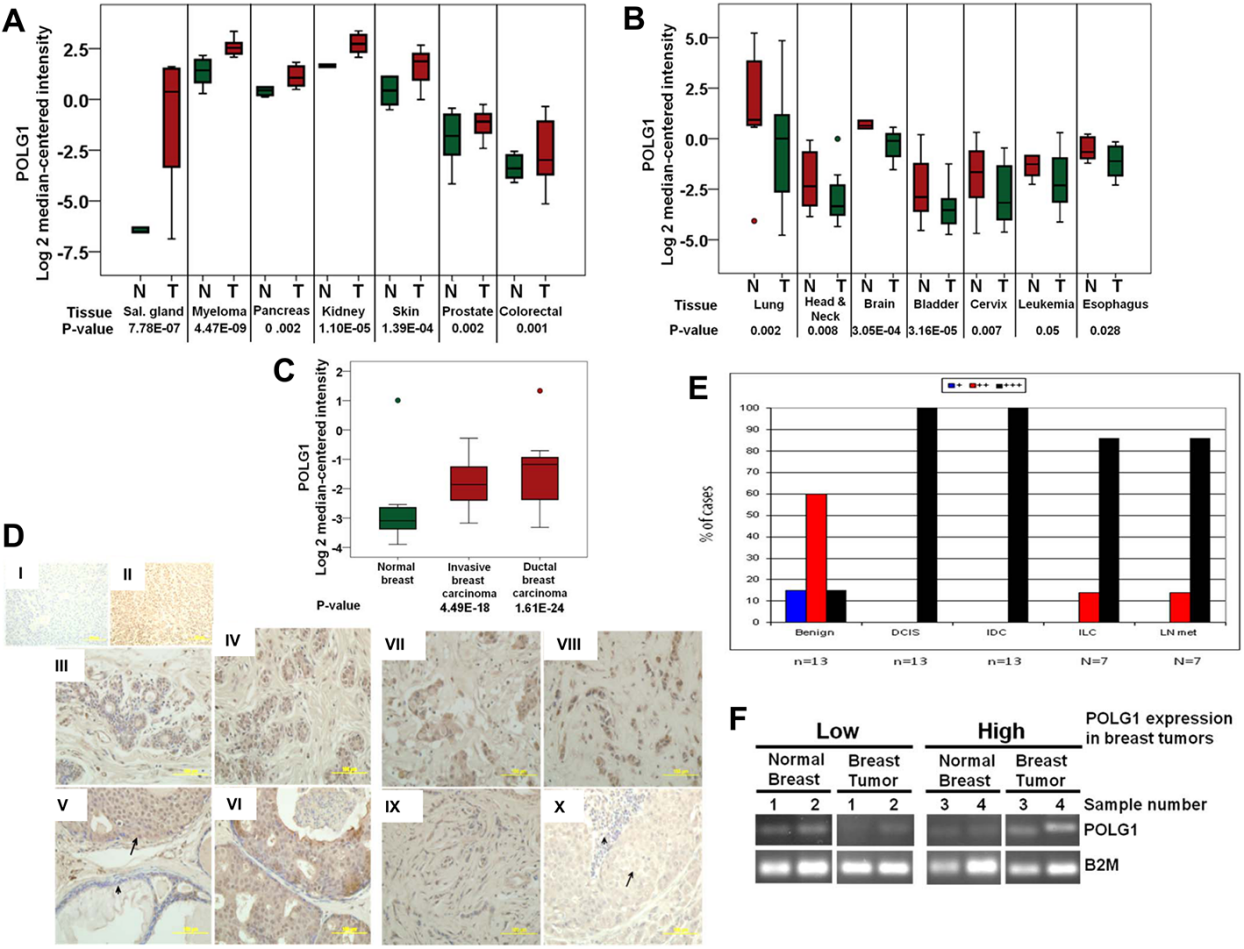
POLG1 expression in primary breast tumors. Oncomine database analysis of POLG1 upregulation (A) and downregulation (B) in normal (N) versus tumor (T) human tissues. **A.** Increased POLG1 expression in salivary gland, myeloma, pancreas, kidney, skin, prostate and colorectal cancer. **B.** Decreased POLG1 expression in lung, head & neck, brain, bladder, cervix, leukemia and esophageal cancer. **C.** Oncomine database analysis of POLG1 expression in human breast cancer. POLG1 expression is significantly increased in both invasive and ductal breast carcinoma compared to normal breast tissues. **D.** Immunohistochemical (IHC) analysis of POLG1 expression in benign breast tissue, breast carcinoma and metastatic carcinomas in regional lymph nodes on tissue array containing 53 breast tissue cores with an anti-POLG1 antibody. **I & II.** Benign liver tissue incubated with (**II**) and without (**I**) POLG1 antibody serving as positive and negative control, respectively. **III & IV.** Representative non-neoplastic breast tissue from patients without and with breast carcinomas, respectively. **V & VI.** Representative low and high grade ductal carcinoma in situ (DCIS), respectively. Notice high expression of POLG1 in DCIS (arrow) compared to low level of expression in non-neoplastic ducts (arrow head). **VII & VIII.** Representative low and high grade invasive duct carcinoma (IDC) cases, respectively. **IX.** Representative invasive lobular carcinoma (ILC) case. **X.** Representative metastatic breast carcinoma in regional lymph node (LN met). Notice high expression of POLG1 in metastatic tumor (arrow) compared to low level of expression in lymhocytes (arrow head). POLG1 protein was visualized using DAB with hematoxylin counterstain. **E.** Graph representing the percentage of different immunohistochemistry score of immunoreactivity for POLG1 expression in benign breast tissue, DCIS, IDC, ILC, and LN met. IHC analysis was done on tissue array containing 53 breast tissue cores with anti-POLG1 antibody. Note high expression (score +++) of POLG1 protein in DCIS, IDC, ILC, and LN met compared to benign breast tissues. **F.** Representative gel pictures from RT PCR study of *POLG1* mRNA expression in a different set of control breast and breast tumor samples. Decreased expression of *POLG1* mRNA in two breast tumors and increased in other two breast tumors compared to normal breast tissues are represented here.

We conducted in silico POLG1 expression analysis in different human cancers. We observed variability in the expression of POLG1 in a variety of human cancers. While POLG1 expression was significantly upregulated in several cancers including salivary gland, myeloma, pancreas, melanoma, prostate and colorectal cancers (Fig 1A), its expression was significantly decreased in lung, head and neck, brain, bladder, cervix, leukemia and esophageal cancer (Fig 1B).

We observed significantly increased expression of POLG1 in both invasive and DCIS compared to normal breast tissue (Fig 1C). To validate this observation, we analyzed POLG1 breast expression in 53 (13 non-neoplastic and 40 neoplastic breast tissue) human breast tissue samples. Tissue microarray slides with several breast tissue cores from different subjects helped us to screen a relatively large number of cases under the same experimental conditions. Examination of non-neoplastic breast tissues from subjects with and without breast cancer revealed expression of POLG1 in < 10% of ductal epithelial cells (score +) in 2/13 cases, score ++ in 7/13 cases and highly expressed (score +++) in 4/13 cases (Table 1) (Fig 1D-III, IV and 1E). POLG1 is highly expressed (score +++) in all cases of DCIS (13/13) (Fig 1D-V and 1D-VI), 5/7 cases of low-intermediate grade IDC (Fig 1D-VII), all cases of high grade IDC (6/6) (Fig 1D-VIII), all cases of ILC (7/7) (Fig 1D-IX) and all metastatic breast carcinomas in regional lymph nodes (7/7) (Fig 1D-X and 1E and Table 1). Only two cases of low grade IDC expressed POLG1 in less than 50% of cells (score ++) (Table 1).

**Table 1:** POLG1 immunohistochemistry in benign breast ductal epithelium (n = 13) and neoplastic breast tissue (n = 40).

IHC Score^a^	Benign tissue	DCIS	IDC	ILC	Lymph node met
+	2 (15%)	0	0	0	0
++	7 (54%)	0	2 (15%)	0	0
+++	4 (31%)	13 (100%)	11 (85%)	7 (100%)	7 (100%)
Total	13 (100%)	13 (100%)	13 (100%)	7 (100%)	7 (100%)

^a^Semi-quantitative scoring of immunoreactivity where

+, < 10% positive cells;

++, 10–50% positive cells;

+++, > 50% positive cells

These findings document high expression of POLG1 (score +++) in 95% (38/40) of neoplastic breast tissue (DCIS, IDC, ILC and LN met) compared to 31% (4/13) high expression in benign ductal epithelial breast tissue (Table 1 and Fig 1E). Negative control sections incubated with secondary antibody did not show any reaction (Fig 1D-I) and liver section incubated with POLG1 demonstrates strong positivity (Fig 1D-II). Our findings also document that there is no correlation between tumor grade and POLG1 expression.

We also analysed *POLG1* mRNA expression in another set of non-neoplastic control breast tissues (n = 10) and breast tumors (n = 10). We observed decreased mRNA expression of *POLG1* in some tumors while in others it was increased compared to non-neoplastic control breast tissues (Fig 1F). These studies suggest that POLG1 expression is significantly altered in a variety of human cancers.

### Frequent *POLG1* Copy Number Variations in Primary Tumors and in Cell Lines

**Fig 2:**
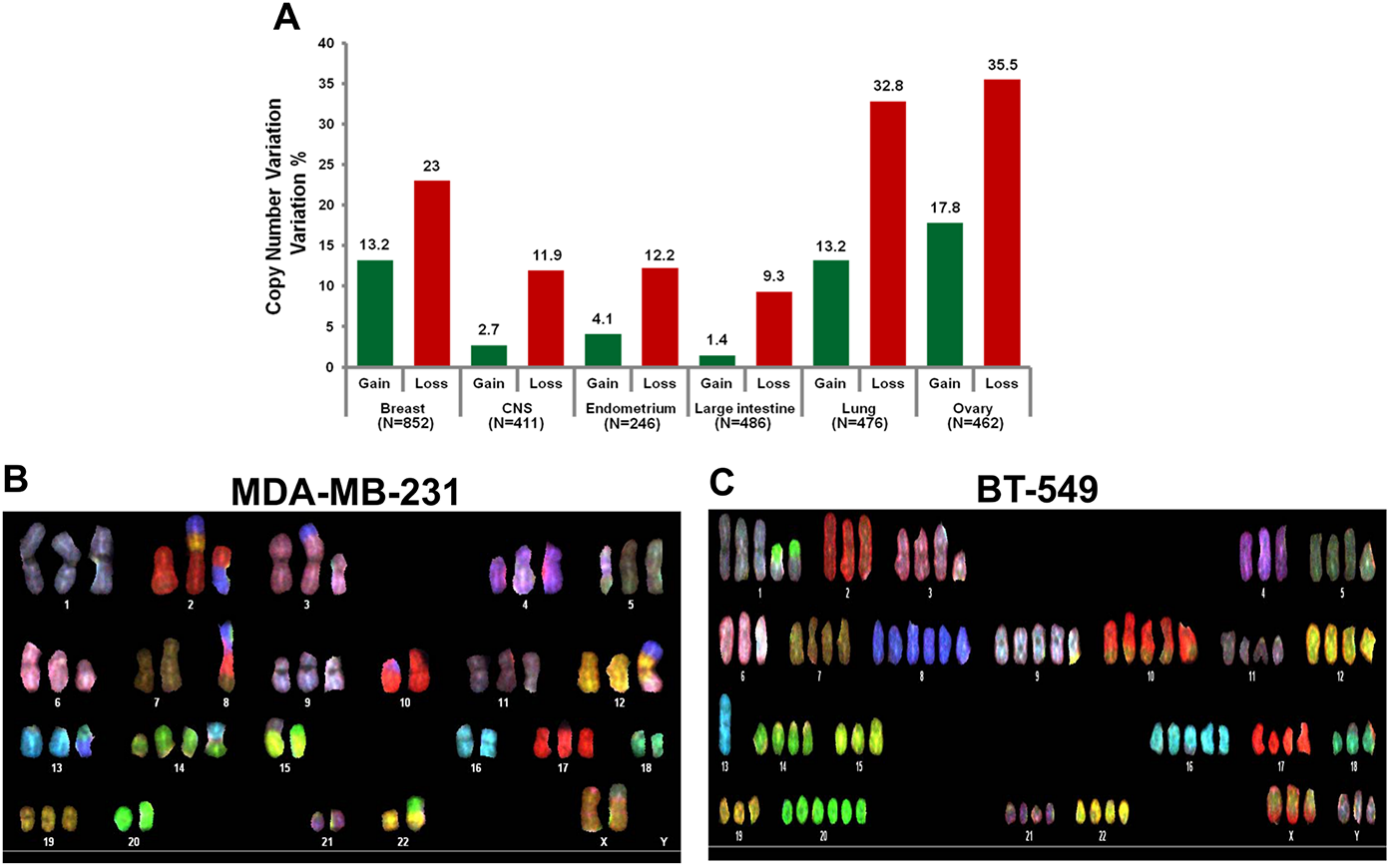
Frequent *POLG1* copy number variations in primary tumors and in cancer cell lines. **A.** Human cancer data from Cosmic database was analyzed for CNVs in *POLG1* gene on February 19, 2014. Gain or loss of *POLG1* copy numbers in different human cancers are represented as % variation. **B & C.** Spectral Karyotyping results from MDA-MB-231 (**B**) and BT-549 (**C**) are represented. In MDA-MB-231 cells, a chromosome translocation involving chromosome 15 is observed, while in BT-459 cells line two normal chromosome 15 and an extra copy of the *POLG1* gene is identified.

**Fig 3:**
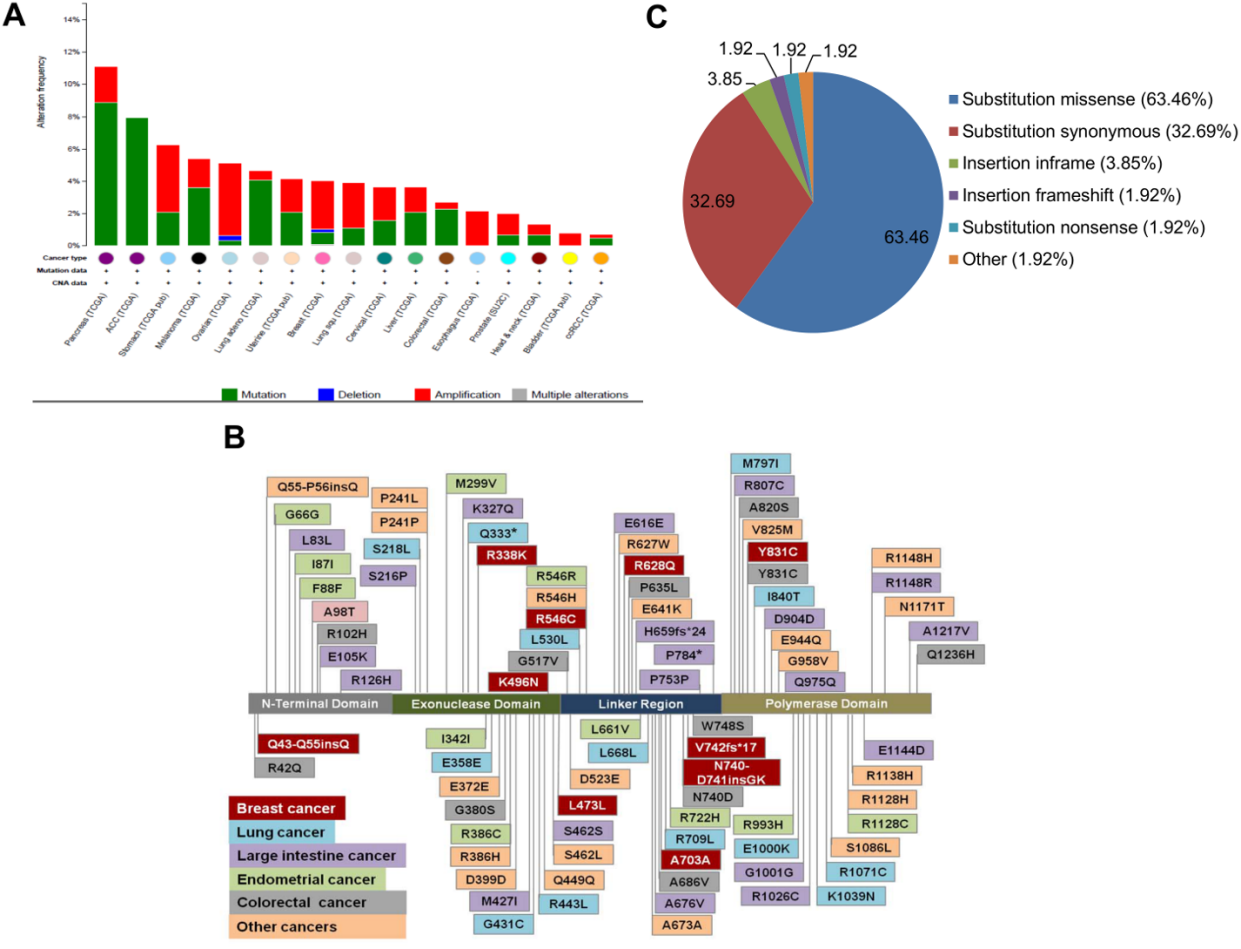
*POLG1* is frequently mutated in primary tumors. **A.** Distribution of different types of variations such as mutations, deletions and amplifications in *POLG1* gene in different human cancers were analyzed using cBioPortal database and represented here as a bar graph. **B.** All the reported somatic mutations in human POLG1 in different human tumors were analyzed from Cosmic database as well as from published studies [21,22] and a schematic showing location of these tumor somatic mutations in human POLG1 protein is presented here. **C.** Distribution pattern of different types of somatic mutations in POLG1 in human cancers was analyzed using Cosmic database and is presented here as a pie chart.

Copy number variations (CNVs) are somatic changes to chromosomes that result in gain or loss of sections of DNA and thus alter functions of the genes present on that section of the chromosome. Changes in the copy number of tumor suppressor genes/oncogenes are prevalent in many types of cancer [33]. We analysed *POLG1* CNVs in breast and other human cancers from two databases: Cosmic database and cBioPortal database (Figs 2A and 3A). Consistent with POLG1 expression data (Fig 1), we observed variability in copy number (both amplification and deletion) of *POLG1* gene in several human cancers including breast, lung, ovary, pancreas, stomach, melanoma, ovary, uterine, cervical, colorectal, prostate, head & neck, bladder and renal carcinoma (Figs 2A and 3A). In our previous analyses, we observed severely reduced mtDNA content and OXPHOS activity in breast cancer cell lines with least mtDNA content and OXPHOS activity in MDA-MB-231 cells [22,34]. Polymerase gamma is involved in replication and repair of mtDNA. We envisioned that alteration of mtDNA in breast cancer cells might be due to changes in the chromosomal copy number of *POLG1* impacting expression of *POLG1* gene. To address this possibility, we carried out chromosomal fluorescence in situ hybridization (FISH) and spectral karyotyping (SKY) analysis of two breast cancer cell lines MDA-MB-231 and BT-549. *POLG1* gene is located on chromosome 15 in humans. In MDA-MB-231 cells, we identified a chromosome translocation involving chromosome 15 and an unidentified partner which carries a single copy of *POLG1* (Fig 2B). MDA-MB-231 cells also contain a normal copy of chromosome 15 which carries a single copy of *POLG1* as well (Fig 2B). While BT-459 cell line showed two normal chromosome 15 carrying the *POLG1* gene (Fig 2C). In addition, a marker chromosome involving a translocation between chromosome 15q and an unidentifiable partner also revealed an extra copy of the *POLG1* gene (Fig 2C). This data indicate that gene copy number variation as well as translocation occurs at the *POLG1* locus in the chromosome.

### *POLG1* Somatic Mutations in Primary Tumors

Our group as well as others have reported mutations spanning the complete *POLG1* gene, including N-terminal, exonuclease, linker and polymerase domain in human cancers [21,22]. We observed high mutation frequency in *POLG1* gene in different cancers (Fig 3A and 3B). *POLG1* somatic mutations identified in databases as well as reported by us and others [21,22] are summarized in Fig 3B. These somatic mutations include nonsense, missense, and synonymous substitutions, inframe insertions, and frameshift mutations (Fig 3C). Interestingly, majority of the mutations in *POLG1* gene in human cancers falls in two categories: missense substitutions (~63%) and synonymous substitutions (~34%). Relative distribution of various mutations is summarized as a pie chart in Fig 3C.

### *POLG1* Germline Variants in Population

Our previous study revealed that mtDNA content is low in African-American when compared to Caucasian-American [35]. Since catalytic subunit POLG1 of polymerase gamma performs critical function in mtDNA replication and repair, dysfunctional POLG1 can impair integrity of mtDNA causing reduction of mtDNA, we were thus interested to analyze if the observed low mtDNA content, has any association with germline variations in *POLG1* gene, and is there any link between the differences in allelic frequencies of variant *POLG1* among the two American populations with different ancestry.

To get better insight on the underlying mechanism of inter-ethnic differences in two large population groups i.e., African-American and European-American, we analyzed the altered allele frequency (mutated allele frequency) of *POLG1* with two distinct databases, i.e., Exome Aggregation Consortium (ExAC) and 1000 Genome database. Interestingly, we identified a missense variation at 1143 amino acid position in the polymerase domain of *POLG1* leading to a change in glutamic acid to glycine, with very striking altered allele frequency between these two population groups. The association of this germline variation with several human mitochondrial diseases is well known [7,36–39]. Sequence homology analysis in polymerase domain of *POLG1* shows that Glutamic Acid at 1143 is evolutionary conserved (S1 Fig), thus any amino acid change at this location may impact the function of polymerase gamma. A comprehensive race based analysis showed more than six times higher allele frequency of E1143G in European-American population (sample size of 33,307 individuals) when compared with African-American population (sample size of 5,192 individuals). The exact altered allele frequency in European- and African-American population was 0.04095 and 0.00625, respectively. Interestingly, 1000 Genome database analyses revealed twenty five fold increase in E1143G allele frequency in European-Americans compared with African-American population. The altered allele frequency in European- and African-American population was 0.03777 and 0.00151, respectively.

We also identified altered frequency of mitochondrial disease associated one variant each from exonuclease and liker region of POLG1: T251I (exonuclease domain of POLG1) and P587L (linker region) [36,40–42] in African- and European-American population. Data comparison through ExAC database showed more than 1.73 times higher altered allele frequency of T251I in European-American population compared to the frequency in African-American population. The allele frequency of T251I in European- and in African-American was 0.00295 and 0.001705, respectively. Similarly, the frequency of P587L was also differentially altered in African- and European-American population. The altered allele frequency in European-American population is 1.7 times higher compared to African-American population. The allele frequency of P587L in European- and in African-American was 0.00289 and 0.0016967, respectively.

### Functional Analyses of *POLG1* Variants

**Fig 4:**
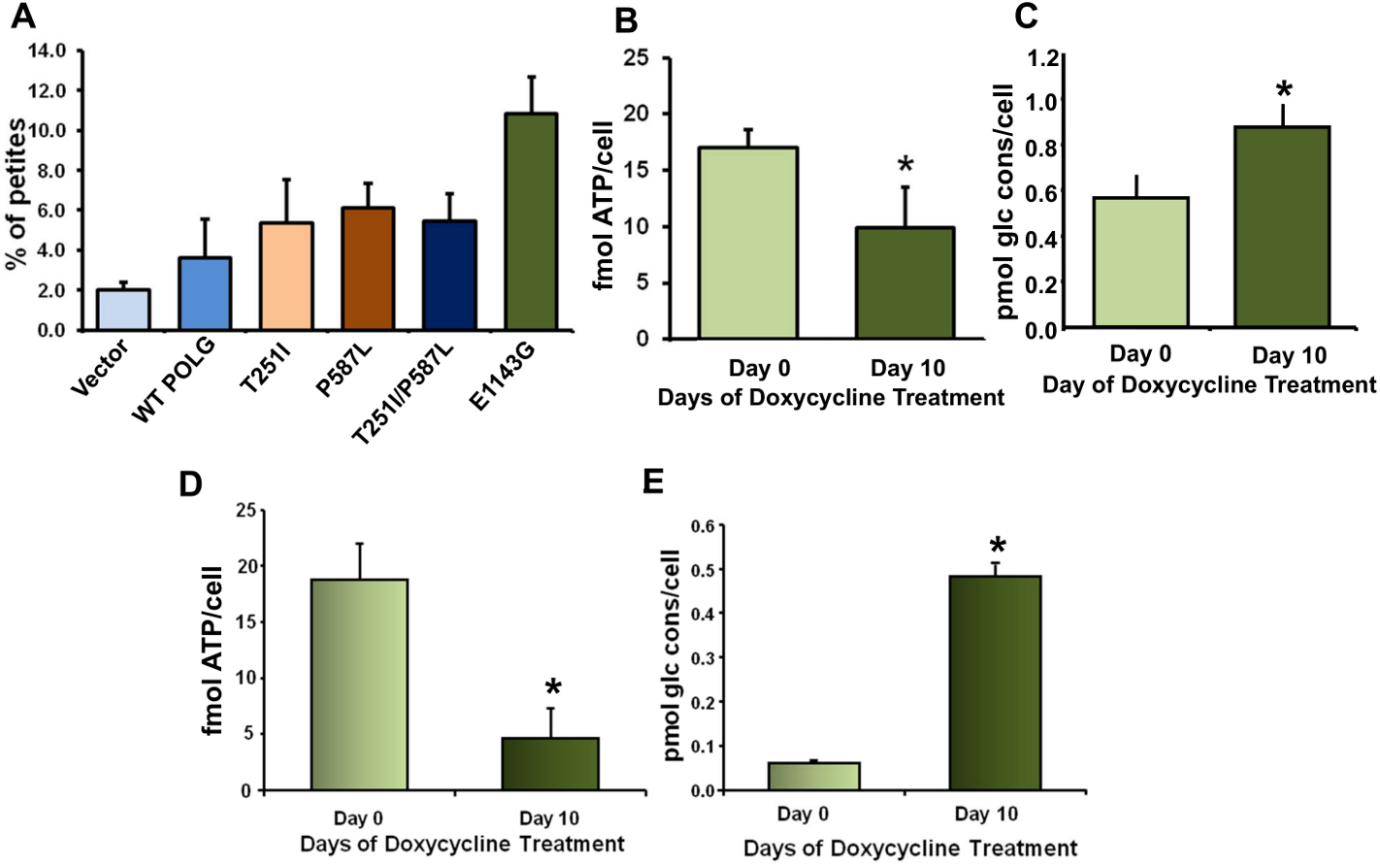
Functional analyses of *POLG1* variants and mutations. **A.** Mitochondria-specific functionality of disease causing human POLG1 germline variants E1143G, T251I and P587L as well as double mutants (T251I/P587L) was analyzed using yeast petite formation assay. Individual expression as well as expression of double mutants in yeast increase petite mutants formation. **B.** Cellular ATP levels in POLG1 E1143G stable cells treated with 1000 ng/ml dox for 10 days. **C.** Glucose consumption in POLG E1143G stable cells treated with 1000 ng/ml dox for 10 days. **D & E.** Functionality of a site directed mutation (D1135A) in the conserved region of the polymerase domain of human POLG1 was analyzed. ATP levels (**D**) and glucose consumption (**E**) in POLG1 D1135A stable cells treated with 1000 ng/ml dox for 10 days. Bars represent the mean ± s.d. *P < 0.05; Student's t-test.

We identified several fold difference in the distribution of germline E1143G mutation frequency among American ethnic populations. Human POLG1 at and around 1143, 251 and 587 amino acid position is evolutionary conserved (S1 Fig). We therefore envisioned that the germline E1143G variation might be associated with altered mitochondrial functions and cellular metabolism. To observe the effects of POLG1 E1143G variant as well as other POLG1 germline variations (T251l, P587L and T251l/P587L double mutation) on mitochondrial functions, we performed yeast petite formation assay. Petite clone formation in yeast is an indication of mitochondrial respiratory chain defects. We cloned human wild-type POLG1, POLG1 E1143G, POLG1 T251l, POLG1 P587L and POLG1 double mutant T251l/P587L in p426ADH yeast constitutive expression vector and transformed these constructs in BY4741 yeast strain individually and let them grew in YPD media for clone formation. All the tested POLG1 mutations increased petite clone formation (Fig 4A). Expression of POLG1 E1143G showed very high petite formation capability indicative of increased mitochondrial defects (Fig 4A).

Next, we stably transfected dox-inducible mutated POLG1 E1143G in MCF-7 cells. We measured cellular ATP levels in POLG1 E1143G stable cells treated with dox for 10 days. We determined a ~2 fold decrease in ATP production in POLG1 E1143G expressing cells (Fig 4B). Further, we measured glucose consumption in POLG1 E1143G cells and observed a significant increase in glucose consumption rate after 10 days of dox-mediated induction (Fig 4C). Increase in glucose consumption indicates a shift from OXPHOS to glycolysis, indicative of Warburg effect, a classical phenomenon seen in cancer cells.

To strengthen our observation that amino acid change/mutation in the polymerase domain of POLG1 leads to alterations in the cellular metabolism and confer tumorigenic properties, we created another site-directed Aspartic acid to Alanine mutation at evolutionary conserved 1135 position (D1135A) near 1143 location in the human *POLG1* polymerase domain (S1 Fig, [22]) and studied the role of D1135A mutation on cellular metabolism and association with tumorigenesis. We measured cellular ATP levels and glucose consumption in POLG1 D1135A stable cells treated with doxycycline for 10 days. Similar to POLG1 E1143G, we observed a significant decrease in ATP production and increase in glucose consumption in POLG1 D1135A cells (Fig 4D and 4E).

### *POLG1* Is Epigenetically Regulated

**Fig 5:**
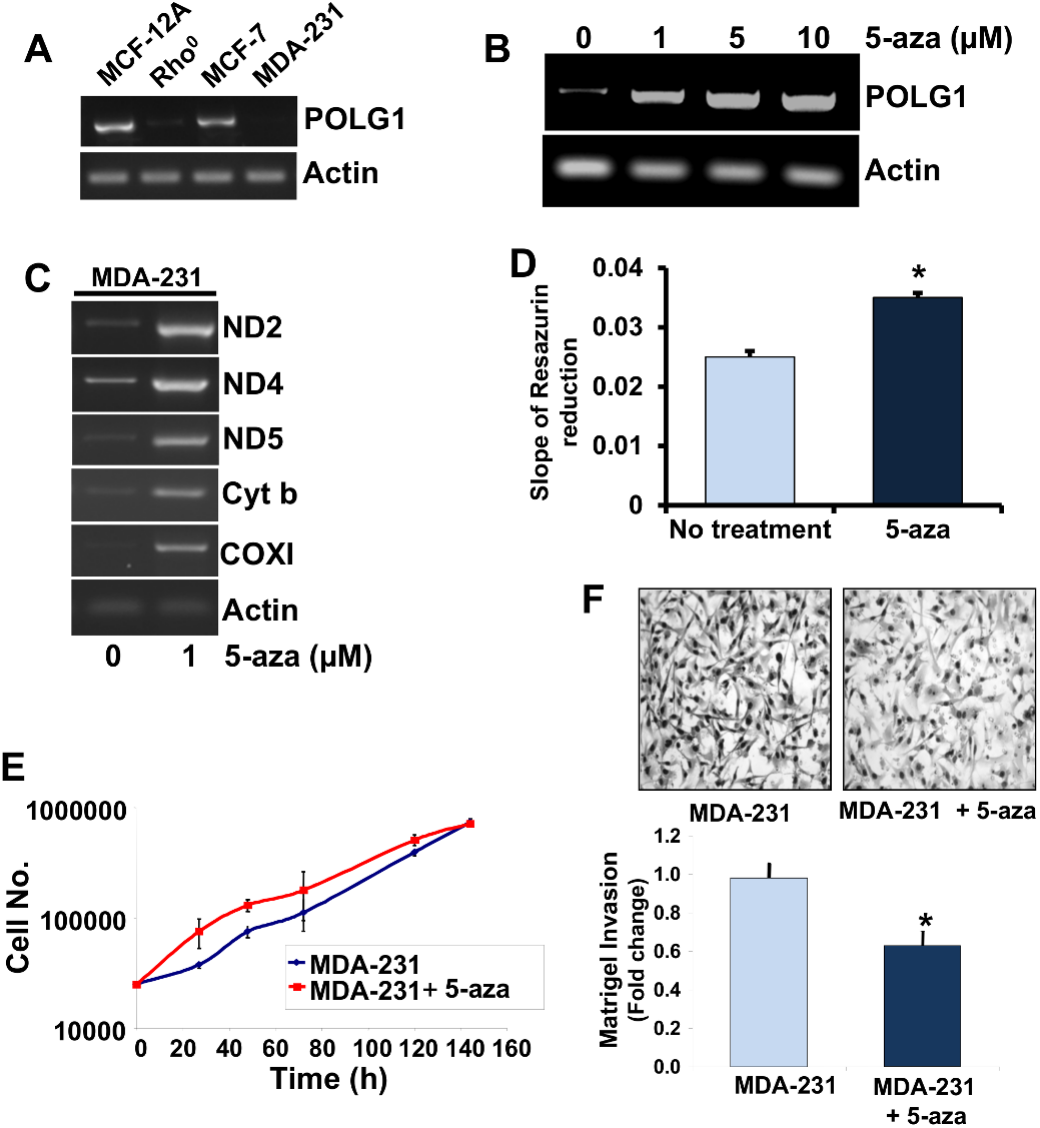
*POLG1* is epigenetically regulated. **A.** RT PCR analysis of *POLG1* gene expression in human breast cell lines and Rho^0^ cells. **B.** RT PCR analysis of *POLG1* gene expression in MDA-MB-231 (MDA-231) cells after 96h of treatment with different doses of 5-aza. Treatment of MDA-231 cells with 5-aza increases expression of *POLG1* in dose-dependent fashion. **C.** Expression of mitochondrial genome-encoded genes after 5-aza treatment (1μM, 96h) of MDA-231 cells. **D.** Increased mitochondrial respiration of MDA-231 cells after 5-aza treatment (1μM) for 96h. **E & F.** Growth rate (**E**) and matrigel invasion capacity (**F**) of MDA-231 cells after treatment with 5-aza (1μM, 96h).

We analyzed mRNA expression level of *POLG1* in non-neoplastic human breast epithelial cells MCF-12A and human breast cancer cell lines MCF-7 and MDA-MB-231, and Rho^0^ (mtDNA depleted) cells. We observed variable expression level of *POLG1* in these cell lines with very low expression in Rho^0^ and MDA-MB-231 cells compared to non-neoplastic breast cell line MCF-12A (Fig 5A) Since *POLG1* expression was very low in MDA-MB-231 cells, we wanted to determine whether the *POLG1* expression is epigenetically regulated in this cell line, therefore, we treated MDA-MB-231 cells with 5-aza, an inhibitor of DNA methylation. Treatment of MDA-MB-231 cells with increasing dose of 5-aza increased expression of *POLG1* in a dose dependent fashion (Fig 5B). 5-aza is a demethylating agent that reduces nuclear genome methylation and increases expression of methylated nuclear-encoded genes [43]. 5-aza treatment increased expression of mitochondria-encoded genes of mitochondrial OXPHOS complex (Fig 5C) as well as mitochondrial respiratory activity measured by the rate of resazurin reduction in these cells (Fig 5D). 5-aza treatment-mediated increase in *POLG1* expression increased growth rate (Fig 5E) but reduced matrigel invasion capacity of MDA-MB-231 cells (Fig 5F). These studies suggest that POLG1 is epigenetically regulated.

### Reversible Tumorigenic Properties Conferred by *POLG1* Variants

**Fig 6:**
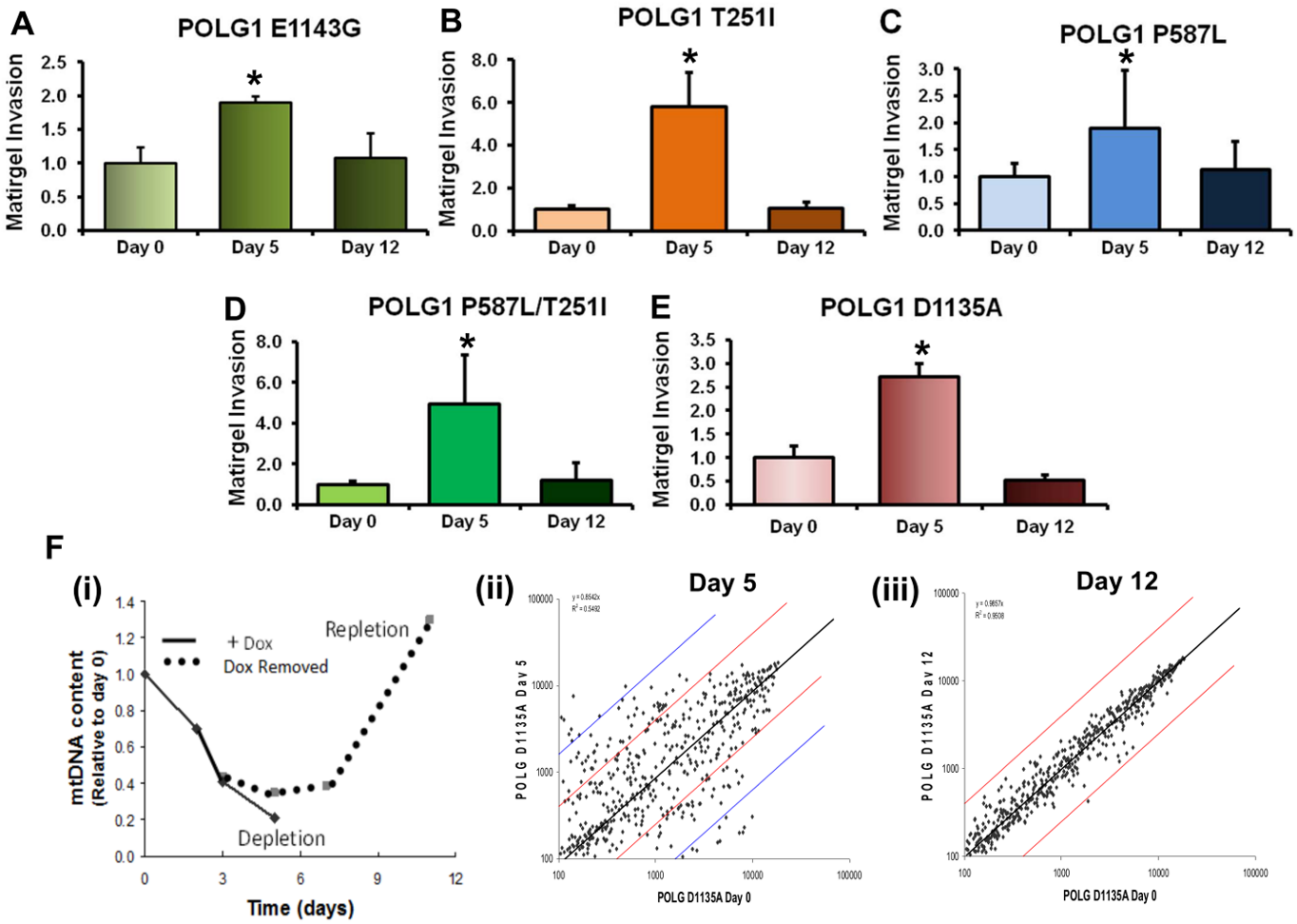
Functional reversibility of *POLG1* variants and mutations. Functional reversibility of POLG1 variants (E1143G, T251I, P587L, T251I/P587L) and mutation (D1135A) was analyzed by performing matrigel invasion assay with MCF-7 Tet-on cells expressing these variants/mutations of POLG1. Cell were treated with 1000 ng/ml dox and sorted for GFP fluorescence. Cells were grown in the presence of dox for 5 days, then the media was changed to dox-free media for 7 additional days. **A-E.** Increased matrigel invasion capacity of POLG1 variants E1143G (**A**), T251I (**B**), P587L (**C**), T251I/P587L (**D**) or mutation D1135A (**E**) after 5 days of dox treatment and reversal of invasion capacity after growing the cells in dox-free media for 7 additional days (day 12). Data represents fold change of invading cells normalized to day 0 ± s.d. *P < 0.05; Student's t-test. **F.** mtDNA content in MCF-7 tet-on cells transfected with POLG1 D1135A after 5 days of dox treatment and in these cells after 7 more days of culturing in the absence of dox (**i**). Illumina human miRNA expression array results from POLG1 D1135A expressing cells after 5 days of dox treatment are represented as a scatter plot (Day 5) (**ii**). POLG1 D1135A cells were then grown in the absence of dox for 7 more days and miRNA expression array results from these cells are also presented as a scatter plot (Day 12) (**iii**).

To analyze the effects of POLG1 E1143G and two other POLG1 germline variants T251l, P587L and POLG1 mutation in polymerase domain D1135A on the tumorigenic properties of cells, we performed *in vitro* matrigel invasion assay. All POLG1 variants used in this assay increased matrigel invasion after 5 days of dox treatment (Fig 6A–6E, [22]). After 5 days of dox treatment, the cells expressing these variants were grown for 7 more days (total 12 days) in dox-free media and matrigel invasion assay was carried out. The cells carrying these POLG1 variants showed decreased matrigel invasion compared to 5 days dox-treated cells (Fig 6A–6E).This indicated that variants of POLG1 under the treatment of dox for 5 days were more invasive, however when the growth media was replaced with media without dox, the expression of mutant POLG1 decreased and a decrease in the cell invasion was observed likewise.

MicroRNAs (miRNAs) add another layer to the epigenetic regulation of the genes [44]. To identify whether miRNAs are involved in the regulation of tumorigenic property induced by *POLG1* mutations, we carried out miRNA-array analysis in cells expressing dox-inducible POLG1 dominant negative allele D1135A [22]. Fig 6F-i demonstrate the depletion and repletion of mtDNA content associated with expression of POLG1 D1135A mutant. We observed more than 4 fold (log 2 ratio) changes in the expression of miRNAs in mtDNA depleted cells at 5 days of induction of POLG1 dominant allele compared to the cells with repleted mtDNA at day 12 (Fig 6F-ii and 6F-iii). Reversal of expression of miRNAs after repletion of mtDNA suggests that reversal of tumorigenic property is associated with repletion of mtDNA (Fig 6).

## Discussion

In our previous report, we identified tumorigenic capability of *POLG1* somatic mutations identified in human breast tumor samples [22]. Although, a large number of *POLG1* germline mutations and their association with several human mitochondrial diseases have been reported, a role for *POLG1* germline variations in pathogenesis of cancer is unknown [7,8,15–18,23,24]. POLG1 is known to carry out mtDNA replication and repair, thus mutation and/or altered expression can affect integrity of mtDNA and mitochondrial function. Therefore, we analysed POLG1 expression, mutations, gene copy number variations as well as epigenetic regulation in human cancers. Importantly, we tested a long-standing question about the role of POLG1 germline variations in cancer predisposition and pathogenesis.

Our analysis using Oncomine database identified highly variable expression of POLG1 in different human cancers (Fig 1A–1C). We determined altered copy number of *POLG1* gene in cBioPortal and Cosmic databases (Figs 2A and 3A). *POLG1* gene was amplified in most of the cancers correlating with increased expression of POLG1 identified in Oncomine database and reported in different cancer types such as pancreas, melanoma, colorectal and renal carcinoma (Figs 1A and 3A). However, in some cancer types such as cervical, esophagus, prostate, bladder and head & neck cancer, *POLG1* gene amplification and expression data do not match each other (Figs 1B and 3A). One of the reasons behind this discrepancy might be the use of two different databases which contain the similar type of cancer data but from different studies and specimens. Another reason might be the presence of *POLG1* mutations that can behave as dominant negative. We have earlier shown that the mutations in the *POLG1* gene may act as dominant negative allele [22]. In this case, even in the presence of amplified *POLG1* gene, the dominant negative allele may ultimately decrease the POLG1 expression in the tumors. Moreover, we identified that *POLG1* is epigenetically silenced in cancer cells. These studies are consistent with methylation of *POLG1* reported recently [43,45,46]. This corroborate with the decreased expression of *POLG1* in cancer even if this gene is amplified. Increased expression of POLG1 in our breast tissue array as well as Oncomine database supports increased *POLG1* gene amplification identified in breast cancer in cBioPortal database. Oncomine database also suggests loss of *POLG1* gene copy number in more human breast cancer samples compared to the gain of *POLG1* gene copy number (Fig 2A). Consistent with Oncomine database observations, our expression analyses show high variability in *POLG1* mRNA expression pattern in human breast tumors (Fig 1F).

Our observations of 5-aza, a demethylating agent, treatment-mediated activation of *POLG1* and thus increased expression of mitochondria-encoded genes and mitochondrial respiration in cancer cells indicate towards hypermethylated status of *POLG1* in cancer cells. A recent report also support our observations about the involvement of DNA methylation and demethylation events in the regulation of *POLG1* in cancer cells [43]. This report also suggests that mitochondrial DNA copy number in cancer cells is strictly controlled by regulation of *POLG1* methylation and demethylation status [43]. Methylation-mediated silencing of *POLG1* in cancer cells also provides a mechanistic explanation to our earlier reports showing decreased mtDNA content in human cancers [22,35]. Moreover, decreased matrigel invasion capacity of cells either upon ectopic expression of *POLG1* (Fig 6A) or upon treatment with demethylating agent (Fig 3F) suggest that increase in expression of *POLG1* is a cancer suppressor event.

Our study suggests a complex regulation of POLG1 in cancer. While gene copy number variation and DNA methylation play role in regulation of *POLG1*, presence of somatic as well as germline variations add complexity to its regulation in cancer. We comprehensively analysed Cosmic database for all the known somatic mutations in POLG1 in different human cancers which is represented in Fig 3B. After further characterization of these somatic mutations, we identified that most of these mutations (~63%) are missense substitutions that lead to a change in amino acid at the particular position in the POLG1 protein (Fig 3C). Our previous report [22] as well as observations from this study suggest that some mutations in the polymerase domain of POLG1 may act as dominant negative and thus can lead to decrease in the mitochondria functions of POLG1.

Synonymous substitution (~34%) is the second major category of mutations occurred in POLG1 in human cancers (Fig 3C). Synonymous substitutions are 'silent mutations' and change the sequence of a gene without directly altering the sequence of the encoded protein. Recent evidences suggest that silent mutations also contribute to human cancer in several ways [47–49]. Silent mutations change the exonic motifs that regulate splicing and are associated with changes in tumor suppressor or oncogene splicing in tumors [50]. Silent mutations can also alter speed or accuracy of mRNA translation as well as mRNA/protein folding [51–53]. Moreover, the natural selection acts widely on synonymous sites [49,51]. Presence of large number of synonymous mutations in *POLG1* indicates a regulatory role of these mutations in cancer.

Mitochondrial depletion syndrome (MDS) results from mutation(s) in nuclear-encoded genes that participate in mtDNA replication, in mitochondrial nucleotide metabolism and in the nucleotide salvage pathway [1,9–14]. So far, only six mitochondrial depletion syndrome (MDS) genes have been identified. These nuclear genes include mitochondrial DNA polymerase gamma, mtDNA helicase twinkle [13], thymidine kinase [10], deoxyguanosine kinase [11], *SUCLA2* [9], and *MPV17*, a mitochondrial inner membrane protein [14]. Of these nuclear genes, *POLG*1 is the most frequent target of mutation and is involved in a variety of mitochondrial diseases. We have earlier shown that somatic mutations in *POLG1* promote tumorigenesis. Since then other reports also support a role for *MPV17* and deoxyguanosine kinase *SUCLA2* mutation induced hepatocellular carcinoma in MDS patients [22,54,55]. However whether germline variations in these or *POLG1* in healthy human population are associated with cancer predisposition remained unclear. MtDNA mutator mice that harbor a mutation in the exonuclease domain (which abolishes the POLG proofreading activity) show a marked reduction in their lifespan because of an increased rate of mtDNA mutation [56]. Interestingly, a recent study reported the development of B-cell lymphoma in mutator mice with different nuclear background [57]. In contrast a study supports significant increased risks of chronic lymphocytic leukemia with increasing mtDNA copy content [58],

We identified a germline variation at 1143 amino acid position in polymerase domain of POLG1 leading to a change in glutamic acid to glycine, with about 25 fold difference in allele frequency between African-American and European-American population. Certain specific populations are known to be more susceptible to cancer with poor survival rate [59–61]. Thus, to have information about the role of *POLG1* germline variations in cancer predisposition, we chose E1143G (POLG1 polymerase domain) and two other T251I (POLG1 exonuclease domain) and P287L (POLG1 linker region) known *POLG1* germline variants and created site-directed point mutations in *POLG1* to achieve these point mutations [22]. We tested the functionality of these three germline variations using yeast petite colony formation assay, an indicator of mitochondrial dysfunction in yeast. As mutations T251I and P587L are often found in *cis* in many mitochondrial diseases, we also determined effect of T251I/P287L double mutant on yeast petite colony formation. Increased yeast petite formation in case of all the three mutations as well as T251I/P287L double mutation suggested the role of these mutations in induction of mitochondrial dysfunction (Fig 4A). To further characterize the tumorigenic potential of these germline mutation, we carried out matrigel invasion assay using cells transfected with dox-inducible constructs of these germline mutations as well as cells containing a site-directed mutation D1135A in polymerase domain of POLG1 [22]. Increased matrigel invasion in case of all the tested mutations (Fig 6A–6E) suggested that these mutations provide tumorigenic properties to the cells through induction of mitochondrial dysfunction. Decreased ATP production and increased glucose consumption in case of POLG1 germline mutation E1143G and another polymerase domain somatic mutation D1135A support tumorigenic properties of POLG1 mutations (Fig 4B–4E).

Causative association of mtDNA depletion with human cancers has been established [62–66]. We have earlier shown that depletion of mtDNA epigenetically regulates nuclear genes and reversal of mtDNA to normal level reverses the epigenetic changes in the nucleus [67]. However, how mtDNA regulates nuclear epigenetic events still remains unknown. We tested the effect of POLG1-mediated mtDNA depletion on nuclear epigenetic changes by use of dominant negative POLG1 D1135A mutation. Here, we show the mtDNA depletion and its effects on nuclear genome in cells after expression of dominant negative POLG1 D1135A (Fig 6F) and the reversal of mtDNA dysfunction-mediated effects on nuclear genome when mtDNA reversed back to the control level (Fig 6). As miRNAs are part of epigenetic machinery of the cells, our observation of altered cellular expression of miRNAs after induction of POLG1 D1135A-mediated decrease in mtDNA and reversal of expression of these miRNAs after removal of dox confirms a role of mtDNA in alteration of nuclear epigenetic mechanisms those are associated with tumorigenic property of the cells (Fig 6F). Our miRNA array studies with D1135A induced mitochondrial dysfunctional genome and normal mitochondrial genome after removal of D1135A effect helped us to conceive that miRNAs might be the possible retrograde signaling mechanism between mitochondrial and nuclear genomes. Additionally, our observations of altered nuclear epigenetic machinery after depletion of mitochondrial DNA due to mutant POLG1 (Fig 6F) and decreased *POLG1* expression (that cause the mtDNA depletion) due to epigenetic silencing of *POLG1* (Fig 5) provide a clue of a possible negative feedback loop between *POLG1* and mitochondrial DNA. In summary, our study provides evidences to show that altered *POLG1* expression as well as germline variations in *POLG1* gene contribute to tumorigenesis.

## Supporting Information

S1 Fig POLG1 protein sequence alignment to show the evolutionary conservation of amino acids at position 251 (exonuclease domain), 587 (linker region), 1135 and 1143 (polymerase domain). (TIF)
