# Supplementary figures and images for: Mitochondrial DNA Polymerase POLG1 Disease Mutations and Germline Variants Promote Tumorigenic Properties
Source: PLoS One. 2015 Oct 15;10(10):e0139846. doi: 10.1371/journal.pone.0139846 (PMC4607296; doi:10.1371/journal.pone.0139846)

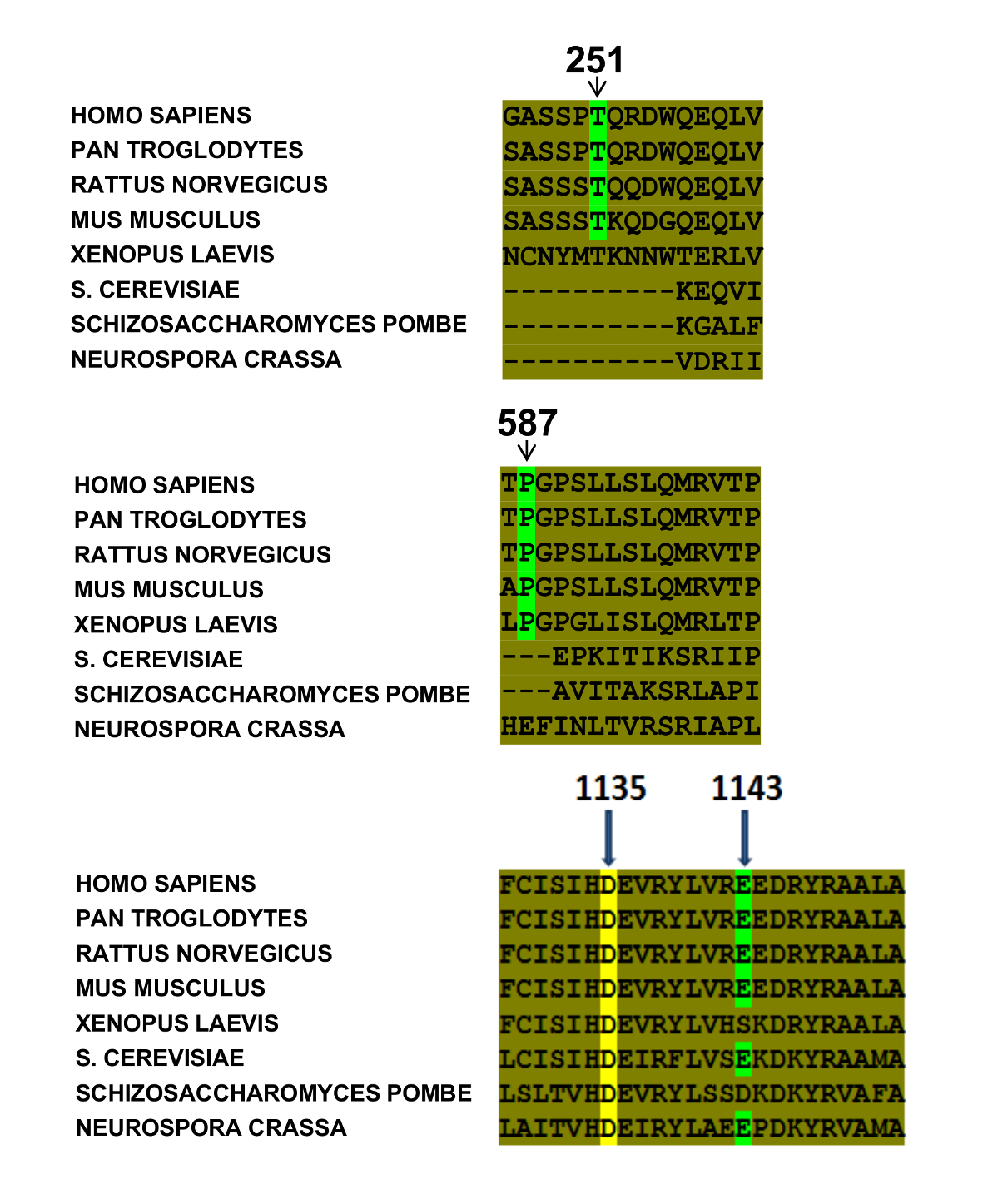

Supplement: S1 Fig — (TIF) [file pone.0139846.s001.tif]
